# Recent progress in enterotoxigenic *Escherichia coli* vaccine research and development

**DOI:** 10.1128/iai.00368-25

**Published:** 2025-11-06

**Authors:** Weiping Zhang, David A. Sack

**Affiliations:** 1 Department of Pathobiology, University of Illinois at Urbana-Champaign 14589, Urbana, Illinois, USA; 2 Department of International Health, Johns Hopkins University Bloomberg School of Public Health 25802, Baltimore, Maryland, USA; University of California at Santa Cruz, Santa Cruz, California, USA

**Keywords:** ETEC, vaccine, diarrhea, platform, infection model

## Abstract

There are no licensed vaccines against enterotoxigenic *Escherichia coli* (ETEC), a group of *E. coli* strains that produce a heat-labile toxin and/or a heat-stable toxin (STa). ETEC is one of the top four leading causes of diarrhea in children in developing countries (children’s diarrhea) and is the most common cause of diarrhea among international travelers (travelers’ diarrhea). Remarkable progress has been achieved in understanding disease mechanisms and developing vaccines against ETEC-associated diarrhea. With an understanding of the disease mechanism and identification of virulence determinants, efforts have focused on developing vaccines that target these virulence determinants using either a cellular (whole-cell) vaccine expressing these antigens or an acellular (subunit) approach that primarily targets ETEC adhesins and/or enterotoxins. However, it remains challenging to develop either a cellular or an acellular ETEC vaccine that effectively protects against ETEC strains and associated diarrheal disease, as ETEC strains produce approximately 30 immunologically heterogeneous adhesins and two distinctive enterotoxins, including the potent and poorly immunogenic STa toxin. Additionally, the prevalence of these virulence factors, particularly adhesins, varies over time and across different geographical regions. In this review article, we summarize the ETEC vaccine candidates that have progressed in the last decade and further discuss the potential challenges associated with the leading candidates. We also highlight the novel epitope- and structure-based multiepitope fusion antigen platform and its application in developing cross-protective multivalent precision vaccines.

## INTRODUCTION

Enterotoxigenic *Escherichia coli* (ETEC) is a group of *E. coli* strains that produce enterotoxins, causing diarrheal disease in animals and humans, particularly in young animals and children (1). ETEC is a leading cause of diarrhea in children younger than 5 years (2–4), as well as the elderly (5, 6), in low and middle-income countries. ETEC is also the most common cause of diarrhea in children and adults from high-income countries traveling to ETEC-endemic regions or countries, including civilian and military personnel deployed in these areas (7–9). ETEC infections are estimated to be responsible for over 200,000,000 diarrhea clinical cases, resulting in up to about 80,000 deaths each year (10–13) and 1,065,000 years of loss due to disability and 6,894,000 years to disability-adjusted life years (14–16). Additionally, ETEC diarrhea is linked to long-term negative impacts in children, including stunting and poor physical growth and cognitive development (17–20). Diarrhea caused by ETEC is often compared to cholera and is considered to be a less severe form of illness, but ETEC can cause diarrhea very similar in severity to cholera and, in fact, was initially discovered as the cause of diarrhea with severe dehydration (21).

ETEC bacteria are transmitted via the fecal-oral route by ingesting contaminated food and water. ETEC strains produce two major types of virulence determinants: adhesin and enterotoxin. ETEC adhesins, including colonization factor antigens (CFAs) and coli surface antigens (CSs), facilitate bacterial attachment to host receptors on intestinal epithelial cells and colonization in the small intestines. Enterotoxins delivered by ETEC bacteria, namely heat-labile toxin (LT) and heat-stable toxin (STa), bind to host receptors GM1 gangliosides or guanylate cyclase C (GC-C), respectively, enter host intestinal epithelial cells via endocytosis, disrupt host cell homeostasis, and stimulate water and electrolyte-rich fluid hypersecretion into the gut lumen, resulting in watery diarrhea and, if it is severe, dehydration and acute death. Thus, ETEC bacterial attachment to host receptors and colonization in the small intestines are the prerequisites for initiating infection. Still, it is the enterotoxins that subsequently play a direct role in causing diarrhea. In contrast to only two enterotoxins, LT and STa (including human-specific STa, hSTa, or STh, and occasionally pig-specific STa, pSTa, or STp [22]), which alone or together are produced by all ETEC strains, there are about 30 fimbrial or non-fimbrial adhesins expressed by various ETEC bacteria (23), though several of these adhesins are more prevalent and are associated with a majority of the clinical cases. Thus, unlike pandemic *Vibrio cholerae,* with which only serotypes O1 and O139 need to be considered, ETEC represents a group of organisms that have different serotypes or pathovars of ETEC that can produce various virulence factors.

The heterogeneity of ETEC bacteria is a key obstacle in vaccine research and development. As an ETEC strain producing any one or two adhesins and either toxin can cause diarrhea in children and travelers, an effective vaccine must protect against the most prevalent and virulent, if not all, ETEC strains or pathovars. However, developing a vaccine to protect against all or even a portion of these ETEC strains has been an unachievable goal historically (reviewed in reference 24). Moreover, the potent 19-amino acid STa toxin (pSTa is an 18-amino acid variant) is poorly immunogenic and has long been believed to induce neutralizing antibodies only when it retains its enterotoxicity (not a safe antigen for a vaccine), but not from a non-toxic STa molecule (25). Therefore, STa has generally been excluded as an ETEC vaccine antigen. However, STa plays a critical role in causing children’s diarrhea and travelers’ diarrhea (26, 27); thus, it would seem that STa must be included in an optimally effective ETEC vaccine (24, 28–30). This dilemma further impedes the development of ETEC vaccines.

Different approaches were attempted to overcome the challenges of ETEC adhesin virulence heterogeneity and the inability to induce protective immunity against the STa toxin with a safe antigen. Since developing a vaccine to cover all ETEC adhesins is not feasible, one strategy is to create a vaccine that focuses on a few adhesins produced by the most prevalent and virulent strains. Clinical and epidemiology studies revealed that ETEC strains producing STa and/or LT toxin and any one or two of the seven adhesins, CFA/I, CFA/II (CS1, CS2, and CS3), and CFA/IV (CS4, CS5, and CS6), are prevalent (31, 32) and are associated with a majority of the diarrheal clinical cases, especially those considered to be moderate to severe cases (31, 33–35). Thus, some or all of these seven ETEC adhesins become the primary antigen targets for developing a cross-protective ETEC vaccine (36–41). Another strategy to overcome virulence heterogeneity is the use of conserved antigens from various ETEC strains for globally protective vaccines (35, 42). A significant breakthrough in ETEC vaccine development in recent years is the identification of STa toxoids and the generation of non-toxic STa toxoid conjugates or genetic fusions as safe antigens to induce antibodies that neutralize STa enterotoxicity and protect against STa toxin-mediated diarrhea (43–45).

This review outlines the recent progress made in ETEC vaccine research and development, particularly since our last review a decade ago (46). Some existing vaccine candidates have progressed to clinical trials. New products aided by novel technologies have overcome historical challenges and advanced rapidly in demonstrating preclinical protection against ETEC intestinal colonization and clinical diarrhea. This article categorizes ETEC vaccine candidates into two categories: acellular vaccines and cellular vaccines. The acellular group includes all potential standalone or combined candidates that use adhesin(s), toxin(s), or adhesin-toxoid compounds as antigens, though a few may be considered as antigens rather than vaccines due to their limited protection coverage. In contrast, the cellular group consists of killed and live attenuated whole-cell products. We track the progress of leading adhesin- and/or toxoid-based monovalent, bivalent, and multivalent acellular ETEC vaccine candidates, as well as multicomponent cellular products (Table 1). In addition, we provide a brief overview of the epitope- and structure-based multiepitope fusion antigen (MEFA) vaccinology platform and infection models, including the controlled human infection model (CHIM) and animal models, and discuss their application in developing ETEC vaccines.

**TABLE 1:** Current ETEC acellular and cellular vaccine candidates^*a*^

Vaccine candidate	Antigen targets	Adjuvant	Route	Status
Acellular				
Adhesin-based				
CFA/I tip adhesin	CfaE	LT_R192G_	ID	Phase 2
CS6 CssBA	CssA, CssB	Double mutant LT (dmLT)	IM	Phase 1
CFA/I/II/IV MEFA	CFA/I, CS1–CS6	dmLT	IM	Preclinical
Noncanonical	EtpA	dmLT, alum	IM	Preclinical
Toxin-based				
LT-STa toxoid fusion	STa, LT	dmLT	IM	Preclinical
dmLT	LT		SL, ID	Phase 1
STa toxoid	STa	dmLT	SC	Preclinical
Adhesin and toxoid-based				
MecVax	STa, LT, CFA/I, CS1–CS6 (CS7, CS12, CS14, CS17, CS21)	dmLT	IM	Preclinical
Adhesin-toxoid	STa, LT, CFA/I, CS1–CS6	dmLT	IM	Preclinical
Combined				
ShecVax	IpaB, IpaC, VirG, GuaB, Stx, Stx2, StxB; STa, LT, CFA/I, CS1–CS6	dmLT	IM	Preclinical
Outer membrane vesicles	*Vibrio cholerae* O1 El Tor; ETEC H10407		IN	Preclinical
Conjugate	*Shigella flexneri* 2a OSP or *Campylobacter jejuni* HS23/36 or HS3 CPS, CfaEB or CSsBA	Alum	SC	Preclinical
Cellular				
Inactivated				
ETVAX	CFA/I, CS3, CS5, CS6, LT	dmLT	Oral	Phase 2b
				
Live attenuated				
ACE527	CFA/I, CS1, CS2, CS3, CS5, CS6	dmLT	Oral	Phase 2b
Combined				
ShigETEC	*Shigella flexneri* 2a; STa, LT		Oral	Phase 1
CVD Shigella-ETEC	CVD 1208 S-122 (*S. flexneri* 2a; CFA/I, LTB)		Oral	Phase 1
	CVD 1233SP::CS2-CS3 (*Shigella sonnei*; CS2, CS3)		IN	Preclinical

^*a*^ The adjuvant and immunization routes listed are from the studies of the most advanced stage. ID, intradermal; IM, intramuscular; IN, intranasal; SC, subcutaneous; and SL, sublingual.

## ACELLULAR ETEC VACCINE CANDIDATES

Acellular ETEC vaccine candidates predominantly target ETEC adhesins and/or toxins. The adhesin-based products include (i) monovalent adhesin tip that is intended to provide cross-protection against a few homologous adhesins and a conserved noncanonical adhesin that is shared among different ETEC strains, (ii) a bivalent chimera composed of one adhesin subunit and another adhesin subunit (or LT components) to expand protective coverage, and (iii) an epitope- and structure-based multivalent recombinant protein for broad protection against several heterogeneous adhesins. The toxoid-based acellular candidates were primarily derived from toxoid mutants of LT in the past and, more recently, from STa toxoids or a combination of LT and STa toxoids intended to protect against both ETEC toxins. By covering a few adhesins and two ETEC toxins, polyvalent adhesin-toxin vaccine candidates further expand protection against homologous strains (those that produce the homologous adhesin and toxins) and heterologous strains (those that express the toxins but different adhesins). While all potential acellular candidates are listed in this section, it is worth noting that some products with coverage limited to only one or two virulence factors are unlikely to be advanced as individual vaccines; rather, they will become antigens in a multicomponent ETEC vaccine.

### Adhesin-based ETEC vaccine candidates

#### CFA/I tip adhesin monovalent and bivalent vaccine candidates

CfaE is the minor tip adhesin subunit of ETEC adhesin CFA/I, the most prevalent member of the class 5 adhesins. ETEC vaccine candidates, derived from the CfaE tip adhesin, including monovalent and bivalent, were developed by the US Naval Medical Research Center (NMRC) to protect against ETEC strains producing CFA/I and other members of the class 5 adhesins, such as CS4 and CS14 (47). In an initial passive protection model, mice intranasally or orally immunized with the monovalent CfaE product (adjuvanted with LT mutant LTK63) developed IgG (serum) and IgA (feces and milk) antibodies, and 78%–93% suckling infant mice born to the immunized mothers survived after lethal challenge with ETEC strain H10407 (CFA/I, LT, and STa) (48). Passive immune protection in mice conceptually proved that a monovalent CFA/I tip adhesin subunit vaccine can protect against ETEC infection.

Passive protection against ETEC infection with anti-CfaE antibody has been confirmed in other studies, as demonstrated by protection from anti-CfaE bovine colostrum against H10407 diarrhea in adult volunteers (48) and non-human primates (49). Also, anti-CfaE human monoclonal antibodies prevented ETEC bacterial attachment and colonization in mouse intestines (50). Subsequent studies using nonhuman primates demonstrated that *Aotus nancymaae* intranasally immunized with CfaE (adjuvanted with LT mutant LT_R192G_, the 192nd arginine of LT replaced with glycine) developed IgG and IgA responses (51), and the immunized monkeys (intranasally immunized with CfaE alone or admixed with the B subunit of LT or cholera toxin, CT) were protected against diarrhea from H10407 (52). In addition to the intranasal route, mice transcutaneously immunized with CfaE also developed antigen-specific serum IgG (53).

A Phase-1 safety and immunogenicity study demonstrated that CfaE was safe but poorly immunogenic in healthy adults when administered subcutaneously (adjuvanted with LT_R192G_). However, when given intradermally, CfaE induced a high rate of serologic responses and antigen-presenting cell responses in volunteers (54). In a following CHIM study, though it was well-tolerated and induced robust antigen-specific immune responses after intradermal administration (adjuvanted with LT_R192G_), this monovalent tip adhesin vaccine candidate did not significantly reduce moderate to severe diarrhea in volunteers challenged with 1–2 × 10^7^ CFUs of H10407, with an efficacy estimated at <28% (55).

CFA/I tip adhesin CfaE was also fused to another adhesin or toxin component and used as a chimera to prepare a bivalent vaccine candidate. Mice intradermally immunized with bivalent protein CfaEB, a fusion of CFA/I major subunit CfaB and tip adhesin CfaE, developed functional antibodies against ETEC H10407 bacterial hemagglutination (56). By substituting the A1 domain of AB_5_ cholera toxin (CT, produced by *Vibrio cholerae*) with tip adhesin CfaE, a holotoxin-structured CfaE-CTA2/CTB chimeric fusion protein was generated and used as the antigen of another bivalent vaccine candidate. Guinea pigs or mice immunized intradermally with this product, or mice immunized intranasally or orogastrically, developed antibodies to CfaE and CT (57, 58). Similarly, the nonhuman primate, *A. nancymaae,* developed strong antigen-specific antibodies after intradermal immunization with the bivalent CfaE-CTA2/CTB. When infected with the H10407 strain, the immunized monkeys had a significant reduction in diarrhea compared to the control monkeys (58). Like the monovalent CfaE, CfaE-CTA2/CTB was also well tolerated by healthy adults and induced antigen-specific antibodies; however, it did not protect against diarrhea after challenge with H10407 (55).

The monovalent CfaE tip adhesin and the bivalent CfaE-CTA2/CTB are the most studied among the acellular ETEC vaccine candidates. However, despite CfaE and CfaE-CTA2/CTB being well-tolerated and immunogenic, they did not protect against ETEC diarrhea in human volunteers challenged with ETEC. Additionally, cross-protection from these two candidates, *in vitro* or *in vivo,* against ETEC bacteria expressing other members of the class 5 adhesins has yet to be demonstrated.

#### CS6 CssBA subunit vaccine candidate

CssBA is another adhesin-based ETEC vaccine candidate (or antigen) developed by the US NMRC and examined for safety and immunogenicity in human subjects. CssBA is a recombinant fusion protein of CssA and CssB, the two structural subunits of ETEC adhesin CS6 (59), and it is designed to protect against the prevalent ETEC strains that produce CS6 adhesin and STa and/or LT toxins. CssBA was shown to be immunogenic in both animals and humans. Mice immunized intradermally with CssBA chimeric protein and adjuvant LT_R192G_ developed serum IgG to each subunit (CssA and CssB) and adhesin CS6, as well as IgA to CS6; mouse serum antibodies inhibited the binding of a CS6 ETEC strain to human colonic cancer cell line HT-29 (59). Similarly, *A. nancymaae* monkeys, after intradermal immunization with CssBA protein and adjuvant double mutant LT (dmLT) (LT_R192G/L211A_; LT mutated at residues 192 and 211), developed serum IgG and IgA antibodies to CS6 and LT. When infected with CS6 ETEC strain B7A (CS6, LT, and STa), the immunized monkey showed no diarrhea. In contrast, 62.5% of the control monkeys developed diarrhea after B7A infection (60).

In an open-labeled Phase-1 safety and immunogenicity trial, healthy adults intramuscularly immunized with three doses of CssBA protein alone or adjuvanted with dmLT showed no severe adverse effects, especially in those vaccinated with CssBA alone or with a low dose of dmLT (0.1 µg). The individuals immunized with CssBA only developed a low IgG response to CS6, but those immunized with the antigen and dmLT adjuvant (0.1 or 0.5 µg) showed increased IgG and IgA responses in a dose-dependent manner (dmLT) (61).

Future challenge studies with the CHIM model will reveal whether this CssBA subunit vaccine candidate can protect volunteers against diarrhea caused by ETEC strains that produce CS6 or other adhesins. However, protection coverage from CssBA would be limited to the disease caused by ETEC strains with the CS6 adhesin. Unless it is combined or co-administered with other ETEC vaccine candidates (or antigens), such as CfaE tip adhesin and dmLT, CssBA can then expand coverage and become a practical ETEC vaccine candidate.

#### Multivalent adhesin CFA/I/II/IV MEFA vaccine candidate

By applying a novel epitope- and structure-based vaccinology platform multiepitope fusion antigen (62), the Zhang laboratory (now at the University of Illinois at Urbana-Champaign) and the Sack laboratory at Johns Hopkins University constructed a polyvalent chimeric protein for broad immunity against the seven most important ETEC adhesins, CFA/I, CFA/II (CS1, CS2, and CS3), and CFA/IV (CS4, CS5, and CS6) (39). This polyvalent protein antigen, termed CFA/I/II/IV MEFA, uses the adhesin CFA/I major subunit CfaB as the backbone, has the less immunogenic B cell epitopes on CfaB substituted by the immunodominant epitopes from the major subunits of the other six adhesins, CS1–CS6, and maintains a protein structure similar to the backbone CfaB (63). Mice intraperitoneally immunized with the polyvalent CFA/I/II/IV MEFA protein (with Freund’s adjuvant) developed robust IgG and moderate IgA responses to the seven target adhesins. Moreover, sera of the immunized mice significantly inhibited the adherence to Caco-2 cells by ETEC and *E. coli* strains expressing any of the seven adhesins (39).

A subsequent study showed that rabbits immunized intradermally or intramuscularly with this adhesin MEFA vaccine candidate (adjuvanted with 1 µg dmLT) equally developed robust IgG antibodies to the seven ETEC adhesins (CFA/I and CS1–CS6) (64). Rabbit serum antibodies inhibited *in vitro* adherence of ETEC or *E. coli* strains that express CFA/I and CS1–CS6 adhesins. More importantly, when challenged with the ETEC strain B7A (CS6, STa, and LT), the immunized rabbits had a two to three log reduction of the B7A bacterial colonization in small intestines, showing a homologous protection as the same from a challenge-rechallenge study in which rabbits were protected from intestinal colonization following an initial challenge with B7A (64).

The Zhang laboratory developed two additional CFA MEFA products and demonstrated their ability to induce cross-protective antibodies against multiple ETEC adhesins. Similar to the construction of the CFA/I/II/IV MEFA (39), a tip adhesin MEFA used CFA/I tip adhesin CfaE as the backbone and integrated the B cell epitopes from the tip adhesin subunits of CFA/I and CS1–CS5, the adhesive subunits of CS6 and CS21, and nonconical adhesin EtpA on the backbone to cover nine ETEC adhesins (41). Mice intraperitoneally immunized with the polyvalent tip adhesin MEFA protein developed robust antibody responses to all nine ETEC adhesins, and the antibodies significantly inhibited *in vitro* adherence of ETEC or *E. coli* bacteria expressing any of the nine target adhesins (41).

The third polyvalent adhesin MEFA protein, also known as CFA MEFA-II, was designed to target the second tier of important ETEC adhesins, including CS7, CS12, CS14, CS17, and CS21 (65, 66). Mice intramuscularly immunized with CFA MEFA-II protein developed robust IgG antibodies to the five adhesins; the derived antibodies significantly inhibited adherence of ETEC bacteria producing any of the five adhesins (65). Additionally, rabbits intramuscularly immunized with a CFA MEFA-II-derived protein, CFA MEFA-IIb, that also carries ETEC toxin components, developed strong antibody responses to the target adhesins and were protected against bacterial colonization in the small intestines (one log reduction) when orally challenged with an ETEC strain expressing adhesin CS21 (66).

These polyvalent CFA adhesin MEFA proteins make it possible for a single immunogen to unprecedentedly protect against all the first-tier or second-tier ETEC adhesins associated with a vast majority of ETEC diarrhea clinical cases, potentially significantly accelerating the development of a safe and broadly protective vaccine against ETEC diarrhea. Their protection against ETEC bacterial colonization in the small intestines was only demonstrated in the studies with the B7A (CS6, STa, and LT) and a CS21 ETEC strain (CS21 and STa) in rabbits. Future studies with ETEC strains expressing other targeted adhesins can verify if they are broadly protective against ETEC intestinal colonization. Additionally, these adhesin MEFAs would not protect against the toxins. Using dmLT adjuvant (-induced anti-LT antibodies) could protect against other LT-producing strains but would not extend to protect against strains that produced STa toxin. Therefore, while they were initially intended to be developed as anti-adhesin vaccines, the adhesin MEFA targeting the first tier of ETEC adhesins, particularly CFA/I/II/IV MEFA, became an antigen for a new multicomponent vaccine candidate.

#### Noncanonical adhesin vaccine candidates

Instead of using the multivalent adhesin-based candidates that are composed of antigens from different adhesins to expand protection coverage, the Fleckenstein laboratory at Washington University at St. Louis searched for noncanonical molecules, including adhesins, and applied the conservative noncanonical molecules as subunit vaccine antigens for broad protection against ETEC (35, 67, 68). PCR screening of 181 ETEC isolates revealed the wide distribution of EtpA, EatA, YghJ, and EaeH among different lineages, but no association with particular pathovars (67). Among the four noncanonical molecules, EtpA and EaeH are putative adhesins. A later study analyzing whole-genome sequence data found that EatA and EtpA were present in 57% and 51.5% of the ETEC strains (*n* = 46) isolated from different geographical locations (35). Serological screening indicated that adult volunteers infected with the ETEC strain H10407 developed antibodies to EatA and EtpA (68), and existing antibodies to these two proteins were associated with a reduction in subsequent ETEC infections (42). Additionally, mice intranasally immunized with EtpA and EatA proteins, either alone or in combination, along with 1 µg of LT adjuvant, developed antigen-specific antibodies, and they showed a significant reduction in bacterial colonization in the small intestine after infection with the ETEC strain H10407, encouraging efforts to develop a subunit vaccine based on nonconical antigens, including adhesin EtpA (67).

A recent study found that mice intramuscularly immunized with recombinant EtpA protein, adjuvanted with dmLT (1 µg) or alum, induced a robust systemic IgG response and a mild to moderate mucosal IgA response. When challenged with an H10407-derivative strain, the streptomycin-treated mice showed a significant reduction in H10407 bacterial colonies in the small intestine. Interestingly, such protection was only observed in mice under the immunization schedule with a 3-week interval, whereas those immunized with a 2-week interval schedule showed no protection (69).

It is encouraging that the intramuscularly administered noncanonical EtpA can induce protective immunity, including mucosal immunity, and, when combined with an adjuvant and administered according to a specific immunization schedule, protects against ETEC colonization in mouse intestines. However, a vaccine composed of EtpA alone would be limited to protection against ETEC diarrhea caused by ETEC strains expressing EtpA (51.5%) (34) and, like other adhesin-based vaccine candidates, would not be adequate due to the lack of antigens against ETEC toxins.

Other potential adhesin-based ETEC vaccine candidates target CS17 (69, 70), CS21 (71), and the noncanonical adhesin EaeH (72), and they are currently in the early stages of preclinical research or development. However, protection coverage from an ETEC vaccine candidate targeting a specific adhesin may not be adequate due to the low prevalence and geographical variation of that adhesin among the diverse ETEC strains.

### Toxoid-based ETEC vaccine candidates

Unlike the adhesins, which are favorable antigen targets in ETEC vaccine development, ETEC enterotoxins, LT, and particularly STa, have largely been excluded as vaccine antigens due to the potent toxicity and poor immunogenicity of STa. LT, or homologous cholera toxin of *Vibrio cholerae*, and mutants with reduced enterotoxicity have been explored as vaccine adjuvants, which can also elicit protective antitoxin antibodies against ETEC LT enterotoxicity. LT and/or STa toxoids have been intensively investigated for potential antitoxin vaccines against ETEC diarrhea in the last decade.

#### Bivalent LT-STa toxoid fusion vaccine candidate

Since ETEC strains produce LT, STa, or LT and STa, an effective antitoxin vaccine must carry antigens of both toxins and induce protective immunity against these two ETEC toxins. To develop an antitoxin vaccine, we must overcome two major roadblocks: (i) the potent toxicity of LT and STa, and (ii) the poor immunogenicity of STa (24). While the non-toxic B subunit of LT (LTB) can be a safe antigen to induce antibodies that block LT toxin binding to the host receptor GM1 through the B pentamer, anti-LTB antibodies do not neutralize LT enterotoxicity, which plays the essential role in causing water secretion from intestinal epithelial cells. On the other hand, STa, a 19 (hSTa) or 18 (pSTa) amino acid peptide, is poorly immunogenic; thus, even if it were not toxic, STa would not induce anti-STa antibodies.

To address the potent toxicity and poor immunogenicity, the Zhang laboratory (then at South Dakota State University, and later at Kansas State University) employed the toxoid antigen approach and a genetic fusion strategy to construct STa-LT toxoid fusion antigens (28). They found that the potent STa toxin abolished enterotoxicity after substituting an individual amino acid of the 11th, 12th, or 13th residue. These full-length STa toxoids retained the native antigenic topology or structure of STa and were recognized by anti-STa antibodies. After being genetically fused to a monomeric LT mutant LT_R192G_ (one mutant LT A subunit fused to one B subunit as a single A_1_B_1_ peptide) at the C terminus, these STa toxoids induced neutralizing antibodies against STa toxin enterotoxicity, and intramuscular immunization of a pregnant sow with a toxoid fusion protected born piglets against STa ETEC diarrhea (28). This study, for the first time, demonstrated that a non-toxic STa molecule (in full-length on a fusion protein) can induce antibodies that neutralize STa enterotoxicity and protect against STa ETEC clinical diarrhea. This finding has laid the foundation for developing toxoid vaccines, particularly STa toxoid vaccines, against ETEC diarrhea.

Subsequent studies from a mini STa mutant library identified several STa toxoids that diminish STa enterotoxicity and retain STa native antigenic propensity (70, 71) and found that genetically fusing an STa toxoid to the N terminus, the C terminus, between the A1 domain and A2 domain, or between the A subunit and B subunit of a monomeric LT toxoid (LT_R192G_) equally induced neutralizing antibodies against STa toxin (29). To further enhance STa immunogenicity, three copies of an STa toxoid were genetically fused to the N terminus, the C terminus, and between the A subunit and the B subunit of a monomeric LT double mutant (mnLT_R192G/L211A_) (72), leading to the construction of a panel of 12 3xSTa_toxoid_-dmLT toxoid fusion proteins (43). These fusions were screened for the ability to induce neutralizing antibodies against STa toxin (and LT toxin as well). It was identified that the toxoid fusion carrying STa toxoid STa_N12S_ (with 12th asparagine substituted by serine), 3xSTa_N12S_-dmLT (later named as 3xSTa_N12S_-mnLT_R192G/L211A_ to specify the monomeric LT_R192G/L211A_ that differs from the AB5 holotoxin-structured LT_R192G/L211A_, dmLT), was optimal in inducing neutralizing anti-STa antibodies (43). Mice immunized intraperitoneally or subcutaneously with toxoid fusion 3xSTa_N12S_-mnLT_R192G/L211A_, adjuvanted with Freund’s adjuvant, SEPPIC ISA51, or dmLT, developed robust IgG antibodies to STa and LT, and mouse serum antibodies neutralized STa and LT enterotoxicity (43, 73). Moreover, antibodies derived from 3xSTa_N12S_-mnLT_R192G/L211A_ protected against STa ETEC diarrhea. Suckling piglets born to the mothers intramuscularly immunized with toxoid fusion protein 3xSTa_N12S_-mnLT_R192G/L211A_ (adjuvanted with dmLT) acquired passive antitoxin antibodies and were protected against clinical diarrhea after challenge with an STa ETEC strain (45, 74). It is worth noting that the protection of this toxoid fusion vaccine candidate against diarrhea in the pig challenge studies was based on passive maternal antibodies (43, 75), and future studies examining the efficacy of active immunity against ETEC diarrhea can better evaluate this toxoid fusion vaccine candidate. Importantly, the antibodies induced by 3xSTa_N12S_-mnLT_R192G/L211A_ exhibited little cross-reactivity with guanylin or uroguanylin. Guanylin and uroguanylin are two STa-like ligands that critically regulate fluid and electrolyte transportation and maintain homeostasis in human intestinal and renal epithelial cells. Cross-reactivity to guanylin or uroguanylin from antibodies induced by ETEC vaccines that included an STa toxoid would have been a potential risk for human health (76).

While the induction of neutralizing antibodies against STa and LT, and protection from STa ETEC clinical diarrhea in animals, constituted a significant breakthrough in ETEC vaccine research and development, the toxoid fusion vaccine candidate 3xSTa_N12S_-mnLT_R192G/L211A_ has yet to be examined in human volunteers for protection against diarrhea, particularly in young children.

However, like the anti-adhesin vaccine candidate CFA/I/II/IV, toxoid fusion 3xSTa_N12S_-mnLT_R192G/L211A_ later became an antigen of a new multivalent vaccine candidate.

#### Monovalent double mutant LT vaccine candidate

Because of the important role it played in ETEC infections, LT, mainly the non-toxic B subunit, has been explored in the development of ETEC vaccines. Antibodies derived from the LT B subunit can block the LTB pentamer binding to host receptor GM1; only antibodies elicited by the A subunit can neutralize the enterotoxicity of the enzymatic A subunit of LT. However, the antibodies from the two LT subunits can act synergistically (77). Early studies of transcutaneous immunization with LT (LT patch) did not demonstrate adequate protection against diarrhea associated with ETEC or other enteric diseases among healthy adults from Germany or the United Kingdom traveling to Mexico or Guatemala (75). Recent efforts focused on the AB_5_ holotoxin-structured double mutant LT (LT_R192G/L211A_). Compared to a single mutant LT, mLT or LT_R192G_, the dmLT showed further enterotoxicity reduction (78). Since dmLT can enhance antigen systemic and particularly mucosal antigenicity, dmLT is mainly explored as an adjuvant for various ETEC vaccine candidates (37, 56, 78–82).

A recent Phase-1 dose-escalation study found that dmLT was safe and well-tolerated up to a dose of 50 µg when administered sublingually or 100 µg orally (82). However, at a regimen of three doses, it induced only moderate anti-LT IgG or IgA. A second Phase-1 dose-escalation study, conducted at a dose range of 0.1–2.0 µg via the intradermal route, concluded that dmLT was safe and highly immunogenic and induced neutralizing anti-LT antibodies (83). The study further revealed that dmLT at a dose of 2.0 µg elicited higher and more prolonged antibody responses. This study suggested that dmLT could be an effective ETEC vaccine candidate (83). While dmLT administered intradermally at a low dose (2.0 µg) induced better serum antibody responses than when given sublingually (50 µg) or orally (100 µg), its ability to induce mucosal IgA was not effective. Less than half of the immunized volunteers showed fecal IgA conversion (83).

Whether anti-LT immunity derived from dmLT can effectively protect against LT + ETEC infections is yet to be demonstrated. A vaccine candidate carrying dmLT as the sole antigen, without adhesin antigens and STa antigen, is unlikely to protect against infections from ETEC strains that produce STa toxin.

#### Monovalent STa toxoid vaccine candidates

Like LT, STa toxin has also been targeted in ETEC vaccine development. Because STa-producing ETEC strains play a more significant role in causing ETEC-associated children’s diarrhea in developing countries (2), once demonstrated for the ability to induce neutralizing antibodies when coupled to a carrier (28), non-toxic STa mutants become the new target for ETEC vaccine development (44, 84). Early studies showed evidence that the poorly immunogenic STa or a shortened STa peptide became immunogenic after being chemically coupled or genetically fused to a protein carrier, such as bovine serum albumin or the B subunit of CT or LT (85, 86). The problem is that for decades, STa toxicity has been believed to be closely associated with immunogenicity, particularly the ability to induce neutralization antibodies (87). This notion, which to a certain degree significantly hampered ETEC vaccine development in the past, shifted after the finding by the Zhang laboratory that, with substitution of one non-cysteine residue, the poorly immunogenic STa toxin can have enterotoxicity diminished but antigenic propensity retained and, when it is genetically fused to a LT toxoid, a resultant full-length STa toxoid can induce neutralizing antibodies against STa toxin (28, 29, 70).

The STa toxoid vaccine consortium, which was led by James Nataro at the University of Maryland and funded by PATH and the Norwegian Research Council, and ETEC research experts from several institutes (University of Maryland, Tulane University, South Dakota State University, Bergen University, and PATH) to develop an STa toxoid vaccine. By cloning the STa gene into a high-copy vector pUC19 and transforming an STa-positive ETEC field isolate with the STa plasmid, the Zhang laboratory produced a recombinant strain for STa toxin production. Using this STa construct and following the protocol that was developed by D. C. Robertson at Kansas State University, the Clements laboratory at Tulane University purified the STa toxin and supplied the toxin to many institutes (including the BEI Resources Repository). In parallel with the construction of an STa mutant mini library and identification of toxoids that retain STa’s native structure and antigenic topology for LT-STa toxoid fusions by the Zhang laboratory (28, 43, 70), a group at the University of Bergen (Norway), led by H. Sommerfelt and P. Puntervoll, synthesized a full library of all possible STa mutants and screened each mutant for toxicity and reactivity with anti-STa antibodies to identify STa toxoids that react to anti-STa antibodies (44). The top 30 STa toxoids were ranked, with most of those having a mutation at residue L9, N12, or A14, and they became potential targets for an STa toxoid vaccine (44).

A pilot study confirmed that STa conjugates, which are chemical conjugates of native STa, either human (STh or hSTa) or porcine (STp or pSTa), with bovine serum albumin, were able to induce neutralizing antibodies against STa enterotoxicity, proving the concept of developing a STa toxoid vaccine (88). However, the antibodies derived from native STa showed reactivity with guanylin and uroguanylin, suggesting STa must be altered to reduce the risk of cross-reactivity with the two GC-C ligands (88). A subsequent study found that STa or toxoid STa_A14T_ coupled to *Acinetobacter* phage coat protein AP205 equally induced neutralizing antibodies against STa enterotoxicity but with lower reactivity to the two homologous ligands (89). The discrepancy in reactivity to guanylin and uroguanylin between the two studies may suggest that the carrier could affect the properties of the anti-STa antibodies. Similarly, a double mutant STa toxoid, STa_L9K/A14T_, coupled to a synthetic virus-like nanoparticle elicited neutralizing antibodies and also showed lower cross-reactivity with guanylin or uroguanylin (90). Additionally, STa double mutant toxoids STa_L9A/N12S_, STa_N12S/A14T_, and STa_N12S/A14H_, as well as triple mutant STa_L9A/N12S/A14T_, induced neutralizing anti-STa antibodies that showed little or no cross-reactivity with either GC-C ligand (91).

The identification of non-toxic STa antigens that induce neutralizing antibodies without cross-reactivity with GC-C ligands, such as guanylin and uroguanylin, overcomes a significant obstacle in developing safe and effective ETEC vaccines. Vaccine candidates based on an STa toxoid antigen alone, however, cannot neutralize LT enterotoxicity and are unlikely to protect against the ETEC strains producing LT alone, though adjuvanted with dmLT at a relatively high dose may lead to protection against LT ETEC strains. Like the dmLT candidate, an STa toxoid vaccine would need to be combined (as an antigen) with other adhesin antigens to create a new multicomponent vaccine against ETEC.

### Toxoid-adhesin multivalent vaccine candidates

It was revealed in the 1970s that an ETEC strain requires the production of an adhesin to colonize the host’s small intestine and a toxin to cause diarrhea (92). Though enterotoxins disrupt host cell homeostasis to cause fluid hypersecretion, adhesin-mediated ETEC bacterial attachment to host receptors and colonization in the small intestines initiate the infection. An early study suggested that with the assistance of anti-adhesin immunity, antitoxin immunity becomes more effective against ETEC diarrhea (93). Therefore, an effective ETEC should induce anti-adhesin immunity to prevent ETEC bacterial colonization and antitoxin immunity to neutralize ETEC toxin enterotoxicity, thus providing synergistic protection against ETEC diarrhea (24, 94, 95).

#### MecVax, a multivalent ETEC vaccine candidate

**Fig 1:**
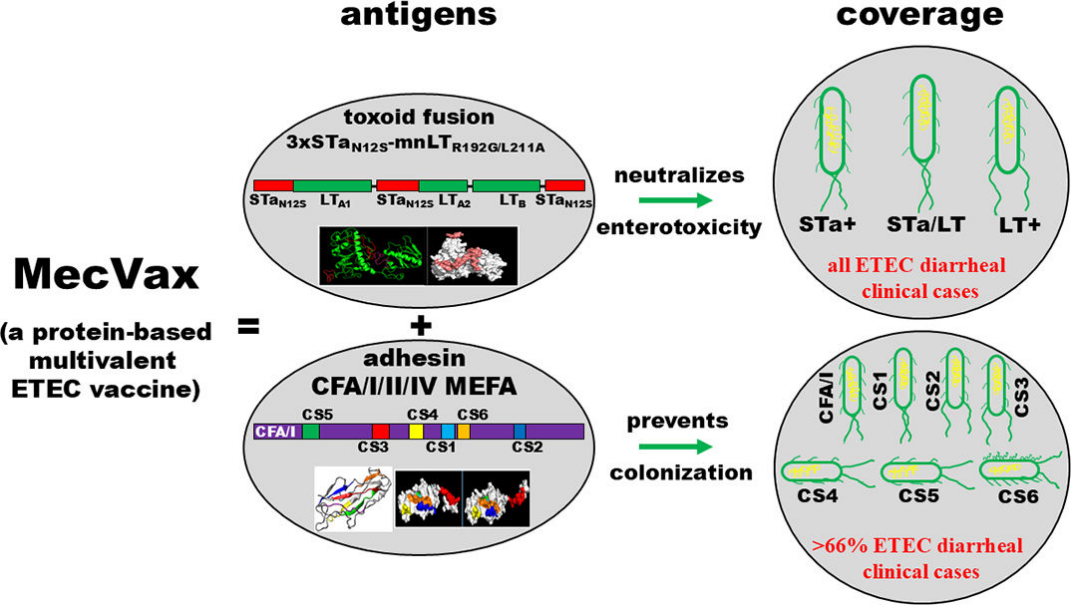
A schematic diagram of MecVax, a protein-based multivalent ETEC vaccine candidate. MecVax is composed of two polyvalent proteins, toxoid fusion 3xSTa_N12S_-mnLT_R192G/L211A_ and CFA/I/II/IV MEFA, constructed with the epitope- and structure-based multiepitope fusion antigen vaccinology platform. MecVax is to protect against children’s diarrhea and travelers’ diarrhea associated with the seven most significant ETEC adhesins (CFA/I and CS1–CS6) and two toxins (STa and LT).

MecVax developed by the Zhang laboratory and the Sack laboratory is composed of two tag-less polyvalent recombinant proteins, CFA/I/II/IV MEFA and toxoid fusion 3xSTa_N12S_-mnLT_R192g/L211A_, which were intended initially for an anti-adhesin vaccine and an antitoxin vaccine, respectively, to protect against the seven most important ETEC adhesins (CFA/I and CS1–CS6) and both ETEC toxins (STa and LT) (96, 97) (Fig. 1). MecVax is designed to induce host anti-adhesin immunity to prevent adherence and intestinal colonization of ETEC strains that produce any of the seven most important adhesins and also induce antitoxin immunity to neutralize the enterotoxicity of both ETEC toxins. Since the two toxins, alone or together, are produced by all ETEC strains, the antitoxin immunity from MecVax is expected to enhance protection (of vaccine anti-adhesin immunity) against strains that produce the seven adhesins (and other homologous adhesins) and one or two toxins, and it should also provide independent protection against the rest of ETEC strains that produce STa and/or LT but different adhesins (other than CFA/I and CS1–CS6), thus all ETEC strains.

Mice co-administered intraperitoneally with tag-less toxoid fusion 3xSTa_N12S_-mnLT_R192G/L211A_ and tag-less CFA/I/II/IV MEFA developed robust antibody responses to the seven adhesins (CFA/I and CS1–CS6) and two toxins (STa and LT); the derived antibodies inhibited adherence of ETEC or *E. coli* strains expressing any of the seven adhesins and neutralized STa and LT enterotoxicity (96). Additionally, both tag-less proteins showed some thermal stability. The CFA/I/II/IV MEFA can be shelved for at least 6 weeks (the entire testing duration) at 37°C or 4 weeks at 50°C, and the toxoid fusion protein showed no visible degradation for 6 weeks at room temperature or 1 week at 37°C (96). While the intraperitoneal route does not apply to human vaccines, this study demonstrated that the two polyvalent ETEC protein antigens can be co-administered to induce broadly functional anti-adhesin and antitoxin antibodies.

The following studies demonstrated that mice immunized intramuscularly or intradermally with toxoid fusion 3xSTa_N12S_-mnLT_R192G/L211A_ and CFA/I/II/IV MEFA developed robust antibody responses to the seven adhesins and two toxins. These antibodies inhibited adherence against the seven adhesins and neutralized both toxins (97, 98). Furthermore, mouse immunization studies were used to demonstrate that these two proteins did not compromise each other in terms of antigenicity, thereby enabling the development of MecVax for synergistic immunity against antitoxin and anti-adhesin (97). Subsequent studies revealed that pregnant sows intramuscularly immunized with MecVax, adjuvanted with dmLT, developed robust IgG (in serum and colostrum) and IgA (in colostrum) antibodies to the seven ETEC adhesins and two toxins. Moreover, suckling piglets born to these immunized mothers acquired antigen-specific maternal antibodies and were protected against ETEC diarrhea after challenge with STa or LT ETEC (97).

MecVax also protects against ETEC bacterial intestinal colonization (99, 100). Adult rabbits intramuscularly immunized with MecVax developed robust IgG antibodies to the seven ETEC adhesins and two toxins. Serum antibodies from the immunized rabbits inhibited adherence from ETEC strains expressing any of the seven adhesins and neutralized the enterotoxicity of both toxins. When challenged orogastrically with ETEC strain H10407 (CFA/I) or ETEC strains expressing CS1/CS3, CS2/CS3, CS3, CS4/CS6, CS5/CS6, or CS6, the immunized rabbits showed over three logs of reduction in ETEC bacteria colonized in the small intestines, compared to the control rabbits (99, 100).

MecVax can be co-administered with another polyvalent protein to expand coverage. Administered together with CFA MEFA-II, which covers 5 additional ETEC adhesins (65), MecVax extended protection against 12 ETEC adhesins (CFA/I, CS1–CS7, CS12, CS14, CS17, and CS21) and two toxins (STa and LT), potentially protecting against the ETEC adhesins that are associated with 86% of ETEC diarrheal clinical cases and nearly all moderate-to-severe cases (101).

Developing MecVax by combining toxoid fusion 3xSTa_N12S_-mnLT_R192G/L211A_ and CFA/I/II/IV MEFA for an effective ETEC vaccine is scientifically and clinically logical. The very encouraging preclinical efficacy against ETEC bacterial intestinal colonization and clinical diarrhea strongly suggests the potential of this ETEC vaccine candidate. However, MecVax has yet to be tested on human subjects; a plan has been developed to obtain an IND approval within 3 years and then proceed to Phase-1 studies. Additionally, further efforts are needed to utilize two animal models to assess the preclinical efficacy of MecVax: a pig model to evaluate MecVax protection against STa or LT toxin-mediated diarrhea, and a rabbit colonization model to assess vaccine efficacy against ETEC intestinal colonization.

#### Adhesin-toxoid vaccine candidate

Instead of combining two polyvalent proteins for the development of MecVax, an alternative approach is to genetically fuse the CFA/I/II/IV MEFA to the toxoid fusion protein 3xSTa_N12S_-mnLT_R192G/L211A_, thereby creating a single antigen for an ETEC vaccine candidate. By replacing a peptide of 131 amino acids on the A1 domain of toxoid fusion 3xSTa_N12S_-mnLT_R192G/L211A_ with the CFA/I/II/IV MEFA, the Zhang laboratory generated the adhesin-toxoid MEFA (CFA/I/II/IV-3xSTa_N12S_-mnLT_R192G/L211A_) (40). This CFA/I/II/IV-3xSTa_N12S_-mnLT_R192G/L211A_ polyvalent protein, adjuvanted with dmLT, induced robust antibodies to seven ETEC adhesins (CFA/I and CS1–CS6) and two toxins (STa and LT) in mice. Mouse serum antibodies inhibited adherence to ETEC or *E. coli* strains expressing the seven ETEC adhesins and neutralized the enterotoxicity of STa and LT. Moreover, pregnant sows immunized intramuscularly with this antigen developed IgG and IgA antibodies to the target adhesins and toxins, and piglets born to the immunized mothers acquired maternal antibodies and were protected against watery diarrhea (89.5% and 80.9%) and any diarrhea (76.5% and 71.4%) when challenged with an STa ETEC or an LT ETEC strain (40).

In a subsequent study aimed at developing a combined vaccine against two groups of enteric bacteria, it was found that CFA/I/II/IV-3xSTa_N12S_-mnLT_R192G/L211A_, while eliciting antibody responses to all target virulence factors, did not induce neutralizing antibodies against STa enterotoxicity, likely due to antigen compromise. Therefore, this adhesin-toxoid ETEC vaccine candidate did not proceed for further development.

Other acellular ETEC standalone vaccine candidates targeting specific antigens, including mucinase YghJ (101, 102) or outer membrane vesicles (OMVs) (103, 104), are currently in the early stages of research and development. Their candidacy required further characterization, at the very least, in preclinical efficacy studies.

### Combined vaccine candidates

While an effective ETEC standalone vaccine is urgently needed, a combined vaccine against ETEC and other enteric bacteria would be more desirable, considering cost-effectiveness and the crowded immunization schedule for children. The current Expanded Programme on Immunization (EPI) is becoming increasingly busy due to the continuous introduction of new vaccines. A combined vaccine would simplify the EPI schedule, reduce the cost of vaccine manufacturing, and improve logistics for vaccine storage, transportation, and clinical administration (102, 103).

#### ShecVax, a protein-based combined vaccine for *Shigella* and ETEC

Since ETEC and *Shigella* are the two most common bacteria that cause diarrhea and infect the same populations, such as children in low- and middle-income countries and international travelers, a combined vaccine for ETEC and *Shigella* becomes more favorable than two standalone vaccines (102, 104).

Assisted with a novel vaccinology platform, the Zhang laboratory constructed a polyvalent *Shigella* MEFA protein immunogen by expressing functional epitopes of *Shigella* virulence factor invasion plasmid antigen B (IpaB), virulence gene (VirG), GuaB, and Shiga toxins 1 and 2 (Stx1A, Stx2A, and StxB) on backbone IpaD and mimicking epitope native antigenicity (105). This polyvalent *Shigella* MEFA protein is broadly immunogenic and induces functional antibodies against the target virulence factors. Mice intranasally immunized with this polyvalent protein developed robust IgG (serum and lung) and IgA (lung and feces) antibodies to all target virulence factors and were protected against lethal pulmonary infections with *S. sonnei*, *S. flexneri* 2a, 3a, and 6 (105).

By combining this *Shigella* MEFA protein with the two polyvalent proteins of MecVax (toxoid fusion 3xSTa_N12S_-mnLT_R192G/L211A_, CFA/I/II/IV MEFA) and confirming antigenic compatibility of the three proteins, we constructed a combined *Shigella* and ETEC vaccine candidate, ShecVax (106). ShecVax contains antigen components derived from the virulence determinants shared by all ETEC and *Shigella* strains; therefore, it is expected to protect against all ETEC pathovars and *Shigella* species and serotypes.

Mice intramuscularly immunized with ShecVax developed robust IgG responses to seven *Shigella* antigens (IpaD, IpaB, GuaB, VirG, StxA, Stx2A, and StxB) and nine ETEC virulence factors (CFA/I, CS1–CS6, STa, and LT); mouse serum antibodies prevented *in vitro* invasion from all four *Shigella* species and the important serogroups, inhibited adherence of ETEC strains expressing any of the seven target adhesins, and neutralized ETEC STa and LT enterotoxicity. Furthermore, mice intranasally immunized with ShecVax were protected against lethal pulmonary infection of *S. sonnei* or *S. flexneri* 2a; rabbits intramuscularly immunized with ShecVax were protected against over 90% colonization in the small intestines by ETEC strain H10407; piglets born to the mother immunized intramuscularly with the combined vaccine candidate were protected against watery diarrhea (100%) when challenged with an STa ETEC strain or an LT ETEC strain (106).

The preclinical efficacy data for this *Shigella*/ETEC combined vaccine are very encouraging. ShecVax can certainly be further improved. While the *Shigella* antigen component (*Shigella* MEFA) induced functional antibodies, the epitopes from some virulence factors may not be optimal based on recent epitope mapping results (107, 108). Additionally, only two *Shigella* species (*S. sonnei* and *S. flexneri* 2a) were tested in the lethal pulmonary efficacy study; future efficacy studies against the other *Shigella* species (*Shigella dysenteriae* and *Shigella boydii*) and the other important serotypes (*S. flexneri* 3a, 6, and 1b), as well as against other ETEC pathovars, and eventually clinical trials are needed to demonstrate ShecVax candidacy against *Shigella* and ETEC infections.

#### An ETEC/cholera OMV vaccine candidate

This combined vaccine candidate for ETEC and *Vibrio cholerae,* developed at the University of Graz, carries outer membrane vesicles from a mutant ETEC strain and a mutant *V. cholerae* strain (109). The mutant ETEC strain is a deletion mutant of a streptomycin-resistant H10407 variant that lacks functional secondary lipid A acyltransferase MsbB (also known as LpxN) and the A subunit gene (eltA) of LT genes (eltAB); the mutant *V. cholerae* strain is a deletion mutant of O1 El Tor Ogawa that lacks the MsbB and the cholera toxin genes (both A and B subunit genes; ctxAB). Mice intranasally immunized with the OMVs of two mutant strains developed IgG and IgA responses to each species. Furthermore, when orally inoculated with wild-type *V. cholerae* or ETEC H10407, infant mice born to dams immunized with the OMV vaccine candidate exhibited at least a three-log and one-log reduction in bacterial colonization in the intestines, respectively (109).

OMVs are primarily based on lipopolysaccharides, which are known to be serotype-specific; thus, an OMV vaccine candidate provides homologous protection but is unlikely to extend protection to heterogeneous serogroups or serotypes. While this ETEC/cholera OMV vaccine candidate was shown to protect against intestinal colonization from two homologous strains, its protection against intestinal colonization by heterogeneous *V. cholerae* serogroups and ETEC strains, and, more importantly, against clinical cholera and ETEC-associated diarrhea, is yet to be demonstrated.

#### ETEC and *Shigella* or *Campylobacter* conjugate vaccine candidates

By conjugating *Shigella flexneri* 2a O-lipopolysaccharide or *Campylobacter jejuni* (HS23/36 or HS3 CPS type) capsule polysaccharide to the ETEC adhesin CFA/I major and tip subunit fusion (CfaEB) or CS6 subunit fusion (CssAB), the US NMRC constructed conjugate antigens to develop bivalent (two conjugates) or trivalent (three conjugates) combined vaccine candidates against ETEC, *Shigella*, and *Campylobacter* (110). Mice immunized subcutaneously with a bi- or trivalent conjugate vaccine candidate developed strong IgG response to ETEC adhesin CFA/I or CS6 and *Shigella* O-lipopolysaccharide and/or *Campylobacter* capsule polysaccharide; sera from the immunized mice exhibited functions against hemagglutination of the CFA/I ETEC strain H10407 (110).

This pilot study demonstrated the feasibility of developing conjugate vaccines against ETEC and other enteric pathogens. However, data from the study are rather preliminary, showing antigen-specific IgG responses and antibody function against hemagglutination by an ETEC strain expressing CFA/I. The study did not examine *in vitro* antibody functions against ETEC adherence, *Shigella* invasion, or *Campylobacter* adherence, nor did it assess preclinical efficacy against diarrhea caused by any of the target bacteria. Additionally, since there are over 30 ETEC adhesins, 59 *Shigella* serotypes, and 47 *Campylobacter* serogroups, these conjugate vaccine candidates, which target one ETEC adhesin, one *Shigella* serotype-specific O-lipopolysaccharide, and two *Campylobacter* serogroup-specific capsule polysaccharides, are unlikely to be cross-protective against the vast heterogeneous serotypes or strains.

## CELLULAR VACCINE CANDIDATES

While acellular vaccines are typically safer, antigenically defined, and often strongly immunogenic in young children in endemic countries or regions, cellular vaccines tend to be more efficient for lasting and local mucosal immunity. A few cellular vaccine candidates, killed or live attenuated, standalone or combined, have been explored against ETEC children’s diarrhea or travelers’ diarrhea (earlier ETEC cellular vaccine candidates were reviewed in reference 24). Recognizing that ETEC strains are immunogenically heterogeneous and that a cellular vaccine candidate derived from a single organism is ineffective, a multicomponent vaccine strategy, involving the combination of a few inactivated or live attenuated strains, has been employed to develop ETEC cellular vaccines for broader protection against various ETEC strains, particularly the prevalent and virulent ones.

### Killed cellular ETEC vaccine candidate

#### ETVAX

Improved from a formalin-killed whole-cell product with the B subunit of cholera toxin (rCTB-CF), ETVAX is currently the most clinically advanced ETEC vaccine candidate, including five Phase-1 studies and two Phase-2b studies (reviewed in references 111, 112). The rCTB-CF developed initially by the Swedish National Bacteriological Laboratory consisted of three strains (O78:H12, CFA/I, STa; O139:H28, CS1; O6:H16, CS2/CS3, and STa) and the recombinant B subunit of cholera toxin (113). This rCTB-CF was tolerated in Swedish adults (36) and children in an ETEC-endemic country when a reduced dose was administered (114) and was shown to reduce diarrheal disease severity among healthy American adults traveling to Mexico and Guatemala infected with ETEC strains expressing LT or LT plus STa, but not against strains that expressed STa only (115). However, rCTB-CF showed no protection against ETEC diarrhea in Egyptian children (116, 117). A subsequent review by a WHO panel recommended “the possibility of improving vaccine efficacy by using a suitable adjuvant or delivery form and/or by increasing the amounts of protective antigens, in particular the CFs, on the bacterial surface should be further explored” (118).

ETVAX (also referred to as MEV early, a multivalent enterotoxigenic *E. coli* vaccine) was developed by the University of Gothenburg and Scandinavian Biopharma in Sweden. ETVAX consists of four inactivated strains and a hybrid protein of ETEC LT B subunit and *Vibrio* CT B subunit (LCTBA) (37). The four inactivated strains are three formalin-killed, toxin-negative O78 *E. coli* strains that overexpress adhesins CFA/I, CS3, or CS5, and one phenol-inactivated K12 *E. coli* strain that overexpresses the CS6 adhesin. Hybrid protein LCTBA is derived from CTB, with seven amino acids replaced by the counterparts of LTB. The initial safety and immunogenicity study with healthy Swedish adults demonstrated that ETVAX, at two oral doses (~2.5 × 10^10^ bacteria each strain; 1 × 10^11^ bacteria in total) with or without adjuvant dmLT, was well tolerated and induced antigen-specific responses. IgA antibodies in lymphocyte supernatant (ALS) responses to LTB, CFA/I, CS3, CS5, and CS6 were increased by 30 to over 100, >3, >10, about 3, and >2-fold, respectively, in the immunized individuals. Fecal IgA response to each antigen was increased by two- to four-fold. However, serum IgG and IgA responses were detected to LTB (about 10-fold) only, but not to the four adhesins (37). A subsequent study with volunteers who had been immunized with ETVAX 13–23 months earlier showed a significantly better rise in IgA ALS responses to the four adhesins and LT after a single dose, indicating that ETVAX induces mucosal immunological memory that can last 1–2 years (119).

ETVAX was immunogenic and tolerated by adults in an ETEC-endemic country (120) and children when a reduced dose was used (121). Bangladeshi healthy adults (*n* = 15) showed no severe adverse effects after oral ingestion of ETVAX, with or without 10 µg dmLT adjuvant. Data from an electrochemiluminescence assay, which was found to be more sensitive than the traditional ELISA, showed that immunized individuals developed ALS IgA responses to four adhesins (CFA/I, CS3, CS5, and CS6) and LTB (without significant adjuvant effect). However, unlike the Swedish adults who developed secretory IgA (sIgA) to all target antigens, the sIgA in the stool samples of the immunized Bangladeshi adults was low and variable; thus, the stool IgA assays were excluded from the study (120). When given at a full (1 × 10^11^ bacteria and 1 mg LCTBA), a half, a quarter, or an eighth adult dose, without or with dmLT adjuvant (at a dose of 0, 2.5, 5.0, or 10 µg), healthy Bangladesh children aged 24–59, 12–23, or 6–11 months showed adverse effects no greater than moderate in severity, with the common adverse effect of dose-related vomiting. Mild fever and diarrhea (in two young groups) were recorded, but the frequency was not significantly different from the placebo group. The 24- to 59-month group immunized with a quarter or half dose without dmLT, or a half dose with any dose of dmLT, showed an ALS IgA increase of 18- to 50-fold responses to CFA/I, CS3, CS5, and LTB, and approximately 8-fold to CS6. Similarly, the 12–23-month group immunized with a quarter or a half dose without dmLT, or a half dose with 2.5 or 5.0 µg (but not 10 µg) dmLT showed a rise of 6–20 times of ALS IgA responses to CFA/I, CS3, and LTB, and about 3–6 times to CS5 and CS6. The ALS IgA responses were low in the 6–11-month group, although significantly greater (for CFA/I, CS3, and LTB) than in the placebo group. The sIgA (to CFA/I, CS3, and LTB) from the fecal samples of the immunized children in the 6–11-month group was also significantly higher (an increase of two times) than in the placebo group. After immunization, plasma IgA levels to CFA/I, CS3, and LTB rose in three age groups, but at lower magnitudes (121).

A similar Phase-1 safety and immunogenicity study was conducted among healthy Zambian adults and children aged 10–23 months and 6–9 months (122). Adults who received one full dose (8 × 10^10^ bacteria and 1 mg LCTBA) and 10 µg dmLT adjuvant showed no differences in the frequency of adverse events (53.3% at least one event of abdominal pain, diarrhea, fever, nausea, and vomiting) compared to the placebo group (40%) and had at least a twofold rise of plasma IgG and IgA to LTB. Children aged 10–23 months showed similar frequencies of adverse events after administration of three doses of an eighth (70% at least one event) or a quarter (80% at least one event) of the full dose (adjuvanted with 2.5 µg dmLT), compared to the placebo group (75% at least one event), and developed plasma IgG and IgA responses to LTB. Children aged 6–9 months also showed no differences in the frequency of adverse effects after receiving an eighth (64.8% at least one event) or a quarter (67.3% at least one event) of the full dose than the placebo group (55.6%) and developed plasma IgG and IgA to LTB. However, antibody responses to the target adhesins (CFA/I, CS3, CS5, and CS6) were not or barely detected in the two children cohorts (122).

Two Phase-2b studies, conducted among Finnish travelers to Benin, West Africa, and children aged 6–18 months in Gambia, further revealed a positive safety and immunogenicity profile of ETVAX (123, 124). There were no significant differences in adverse effects between the immunized group and the control group. Healthy adults from non-ETEC-endemic countries developed serum IgG (a 15-fold increase) and IgA (a 22-fold rise) to LTB, as well as IgG (2.1-fold) and IgA (3.5-fold) to O78 lipopolysaccharide (LPS). However, IgG and IgA to the adhesins were not examined as they were expected to be poor (123). Results from Gambian children aged 9–18 months, who received three doses of vaccine on days 0, 15, and 90, were presented at a scientific meeting in June 2025. Data showed no significant safety concerns in these young children. When excluding coinfections (the primary outcome), the efficacy was approximately 25%. When coinfections were included, the efficacy increased to about 50%, and when all coinfections, except parasites, were included, the efficacy reached about 80%. Given these results, the authors suggested that the vaccine may have had some protective effect against enteric pathogens beyond ETEC (125).

Results from ETVAX Phase-1 and Phase-2b trials are very encouraging. Overall, ETVAX is well-tolerated in healthy adults from ETEC non-endemic countries and endemic countries, as well as in young children from endemic countries, with a reduced dose. ETVAX induces significant ALS IgG and IgA responses in adults to LTB and four adhesins (CFA/I, CS3, CS5, and CS6) (and anti-adhesin antibodies cross-react with CS1, CS14, CS17, and CS7 [126]) and at least LTB in young children in ETEC-endemic countries. Potentially, this vaccine can be administered orally in combination with another vaccine, such as a rotavirus vaccine, to meet a single EPI. It should be noted that ETVAX carries the LT antigens (LCTBA antigen and dmLT adjuvant) but no STa antigens. It remains to be determined whether ETVAX would provide any protection against STa-only ETEC strains based on the adhesins present in the vaccine.

### Live attenuated cellular ETEC vaccine candidate

#### ACE527

ACE527, developed by ACE BioSciences and PATH, is composed of three live non-toxic strains together to produce six ETEC adhesins and recombinant LT B subunit protein: ACAM2022 (CS5/CS6 and LTB), ACAM2025 (CFA/I and LTB), and ACAM2027 (CS1/CS2/CS3 and LTB) (127). This vaccine candidate, administered at doses of 10^10^ and 10^11^ bacteria, was demonstrated to be well-tolerated and immunogenic (to LTB and some adhesins) in healthy adults in a Phase-1 study. A subsequent Phase-2b trial, at a regimen of two doses of 10^11^ bacteria, showed that while it conferred protection against severe diarrhea, with an efficacy of 41%, after challenge with ETEC strain H10407, ACE527 did not demonstrate protection against moderate-to-severe diarrhea (128). Additionally, the high oral dose was linked to gastrointestinal adverse effects.

Using a reduced dose of 10^10^ CFUs and including 25 µg dmLT adjuvant, another trial showed no significant differences in solicited and unsolicited adverse effects. However, gastrointestinal adverse effects tended to be more common among the vaccine groups (with or without dmLT). Volunteers vaccinated with ACE527, with or without dmLT, developed ALS IgA and serum IgG and IgA response to LTB and CFA/I. After challenge with H10407, ACE527 adjuvanted with dmLT conferred 65.9% efficacy against severe diarrhea and 58.5% efficacy against diarrhea of any severity; however, ACE527 without dmLT adjuvant showed no significant protection against H10407 infection (129).

Further analyses revealed that the production of adhesin antigens from the three live strains of ACE527 was deficient (128), resulting in an insufficient immune response to the target adhesins and perhaps only partial protection against ETEC H10407 infection. With these results, the development of this vaccine candidate was discontinued. The lack of other important antigens, including some noncanonical adhesins (130) and STa antigens, may have limited the efficacy of this ETEC vaccine candidate.

### Combined cellular vaccine candidates

#### ShigETEC

ShigETEC is a live attenuated combined vaccine against shigellosis and ETEC diarrhea. ShigETEC, developed by Eveliqure Biotechnologies GmbH, uses a live attenuated noninvasive *Shigella* strain to express an ETEC fusion protein of LT B subunit and an STa toxoid (131). The *Shigella* host strain was derived from S. *flexneri* 2a 2457T by removing its rfbF gene, setBA genes, and invasion plasmid antigen B (IpaB) and C (IpaC) genes. The rfbF gene is involved in the biosynthesis of the O-antigen of lipopolysaccharide. The setBA genes encode an AB5 heat-stable *Shigella* enterotoxin 1 (ShET). IpaB and IpaC are key components of the type III secretion system. The ETEC antigen, a chimeric fusion of LTB and STa toxoid, was generated by inserting STa toxoid STa_N12S_ at the C terminus of the LT B subunit. To enhance ETEC toxin immunogenicity, three tandem repeats of LTB-STa_N12S_ were fused to and expressed by the *Shigella* infA gene in the invasion plasmid (131). After intranasal immunization with ShigETEC, three doses 2 weeks apart, mice developed strong systemic antibodies to the vaccine and LTB but moderate antibodies to STa. Mucosal IgA to the vaccine strain and LTB were also detected, but mucosal IgA to STa was very low. Mouse serum antibodies were able to block LT binding to GM1 receptors and neutralize the enterotoxicity of LT. However, there was no neutralization activity against STa enterotoxicity due to a very low level of anti-STa antibody responses (though antibodies derived from parenteral immunization of LTB-STa_N12S_ neutralized STa enterotoxicity) (131). ShigETEC protected the intranasally immunized mice against lethal pulmonary infections from *S. sonnei* and *S. flexneri* 6 (131).

A Phase-1 study demonstrated that ShigETEC was well-tolerated, showing no severe adverse effects after administration of a single dose ranging from 10^9^ to 2 × 10^11^ CFUs, with mild headaches in two individuals and three cases of reactogenicity (mild diarrhea and mild vomiting in the highest dose group) observed, possibly related to vaccination (132). With a reduced dosage, 5 × 10^10^ CFUs, at two, three, or four doses (3 days apart), ShigETEC was better tolerated (with only five mild reactogenicity events reported from two subjects) and induced a strong serum IgA response to the vaccine strain (lysates). After a single dose, 87.5% of the vaccinees exhibited a four-fold increase in IgA responses, and 100% responded after multiple immunizations. However, serum IgG responses were much lower (132). ALS IgA and fecal IgA responses were detected to the vaccine strain lysates; however, the IgA responses to LTB were much weaker, and no significant STa IgG or IgA response was detected from any of the subjects (132).

While the ShigETEC showed a good safety profile among healthy adults in the Phase-1 trial, it did not induce any measurable antibody responses to the ETEC STa toxin. The inclusion of dmLT adjuvant may enhance antibody responses to LT and potentially to other target antigens as well. Additionally, due to a lack of ETEC adhesin antigens, this vaccine candidate is likely to encounter challenges in combating ETEC bacterial intestinal colonization. Results from the ongoing CHIM study (Safety and efficacy study of ShigETEC; NCT07049159) are expected to provide important information about the candidacy of this combination vaccine.

#### CVD *Shigella* and ETEC multivalent vaccine candidates

The Center for Vaccine Development and Global Health at the University of Maryland is developing a *Shigella*-ETEC multivalent vaccine candidate by using a few live attenuated (GuaAB and ShET genes deleted) *S. flexneri* and *S. sonnei* vaccine strains that each expressed two ETEC adhesins or an adhesin and one toxin (133). This strategy involves using up to five or six attenuated *Shigella* strains as live vectors for the expression of a total of 10 ETEC antigens: CVD 1208S-122 (*S. flexneri* 2a + CFA/I, LTB), CVD 1233SP::CS2-CS3 (*S. sonnei* + CS2, CS3), CVD 1213-312 (*S. flexneri* 3a + CS1, CS5), CVD 1216-420 (*S. flexneri* 6 + CS4, CS6), SVD 1224-501 (*S. flexneri* 1b + CS14, mST), and CVD 1254 (*S. dysenteriae* 1 + Stx1B) (134). These strains (different *Shigella* strains expressing different ETEC antigens) will be initially examined individually for safety, immunogenicity, and efficacy. Then, they will be combined in a mixed formulation for a multivalent *Shigella*-ETEC vaccine to protect against both pathogens (135).

##### CVD 1208S-122 strain

This bivalent *Shigella*-ETEC vaccine strain uses *S. flexneri* 2a strain 1208S as the host to express ETEC adhesin CFA/I, LT A2 domain, and B subunit. Live attenuated *Shigella* vaccine strain CVD 1208S is derived from CVD 1208, a ∆*guaBA*∆*sen*∆*set* mutant of *S. flexneri* 2a (136), by using an animal-material-free medium (137). A single oral dose (10^8^ or 10^9^ CFUs) of CVD1208S was well-tolerated by healthy adults and induced anti-LPS IgA ASC in all immunized volunteers (*n* = 7) but moderate serum IgG and mild fecal IgA responses (137). The insertion of an ETEC CFA/I operon, the LT A2 domain, and the B subunit into the CVD 1208S chromosome results in a new vaccine strain, CVD 1208S-122, intended to protect against *Shigella flexneri* 2a and ETEC strains expressing CFA/I adhesin (138).

Mice intranasally immunized with CVD 1208S-122, 10^6^ CFUs, three doses weekly, developed serum IgG to LPS of *S. flexneri* 2a and to ETEC CFA/I adhesin (anti-LTB IgG was not examined). When challenged with homologous *S. flexneri* 2a or ETEC strain H10407, the immunized mice showed protection against body weight loss and also diarrhea (although no data were included). However, there was no protection against ETEC bacterial colonization in the small intestines (138).

A Phase-1 safety and immunogenicity study (Study of responses with *Shigella*-ETEC vaccine strain CVD 1208S-122; NCT04634513) conducted recently at the University of Maryland at Baltimore showed that CVD1208S-122 has no safety concerns and induces antibody responses to the LPS of *S. flexneri* 2a and ETEC antigens CFA/I and LT (139).

##### CVD1233SP:CS2-CS3 strain

This *Shigella*-ETEC strain was derived from the CVD 1233 strain, a ∆*guaBA*∆*sen* deletion mutant of *S. sonnei* 53G. This deletion mutant strain was initially used to host a plasmid carrying a cistronic ETEC adhesin (CS4) and an LT mutant (LTK63), thereby expressing two ETEC antigens (CS4 and LT), and becoming a *Shigella*-ETEC vaccine strain CVD 1233(pCS4-LTK63). Guinea pigs intranasally immunized with CVD 1233 (pCS4-LTK63) developed IgG (serum) and IgA (tear) antibodies to *S. sonnei* LPS, ETEC CS4 adhesin, and LT toxin; they were protected against keratoconjunctivitis from *Shigella* homologous (but not heterologous) challenge (135).

However, CVD 1233 can spontaneously lose a large virulence plasmid, which is essential for vaccine strain efficacy. An introduction of mutations on the plasmid improved plasmid retention, leading to a stabilized strain, CVD 1233-SP. CVD 1233-SP can induce strong anti-LPS IgG and IgA antibodies in the intranasally immunized guinea pigs and protect against keratoconjunctivitis in the Sereny test (140). Insertions of the CS2 operon and CS3 operon into the chromosome of CVD 1233-SP resulted in *Shigella*-ETEC vaccine strain CVD 1233-SP:CS2-CS3 (140). This new vaccine strain elicited strong IgG (serum; more than a three- or fourfold increase) and IgA (tear; more than a twofold increase) to *S. sonnei* LPS and also strong IgG and IgA responses to CS2 and CS3 in the intranasally immunized guinea pigs. Additionally, serum antibodies from the immunized guinea pigs significantly inhibited *in vitro* adherence to CS2 or CS3 ETEC bacteria. Moreover, when challenged with *S. sonnei* 53G, 80% of the immunized guinea pigs (four out of five) did not develop keratoconjunctivitis (140).

There are a few other *Shigella*-ETEC strains under the early stage of development, including the live attenuated *S. flexneri* 3a strain (CVD 1213-312) to express ETEC adhesins CS1 and CS5, *S. flexneri* 6 (CVD 1216-420) to express ETEC adhesins CS4 and CS6, *S. flexneri* 1b (CVD 1224-501) to express ETEC adhesin CS14 and a STa toxoid, and *S. dysenteriae* 1 (CVD 1254) to produce the B subunit of Shiga toxin (133, 134). The immunogenicity and preclinical efficacy of these candidates have yet to be investigated or published.

While CVD 1208S-122 and CVD 1233SP::CS2-CS3 were shown to be immunogenic and protective against keratoconjunctivitis following homologous challenge, their protection against shigellosis and ETEC diarrhea has not been demonstrated. Mixing five or six bivalent *Shigella*-ETEC vaccine strains certainly expands potential protection against heterogeneous *Shigella* species and important serotypes, as well as the target ETEC strains. However, it could also significantly complicate vaccine formulation, reduce immunogenicity to individual virulence targets, and increase the risk of adverse effects. Additionally, the expression levels of the ETEC antigens by these vaccine strains are to be determined. Suppose the production of ETEC antigens is inadequate, as in the multicomponent cellular ETEC vaccine candidate ACE527 or rCTB-CF. In this case, this *Shigella*-ETEC combined vaccine candidate may exhibit lower immunogenicity to ETEC adhesins and insufficient protection against ETEC diarrhea (130).

## A NOVEL VACCINOLOGY PLATFORM TO OVERCOME VIRULENCE HETEROGENEITY

A new vaccinology platform, termed MEFA (multiepitope fusion antigen), provides an alternative solution to multicomponent vaccines and vaccines based on conserved antigens, thereby overcoming the virulence heterogeneity challenge in ETEC vaccine development. MEFA is an epitope- and structure-based technology that combines epitope vaccine and structure vaccine concepts to generate a polyvalent immunogen for a vaccine that is cross-protective against different virulence factors, strains, or diseases (62).

**Fig 2:**
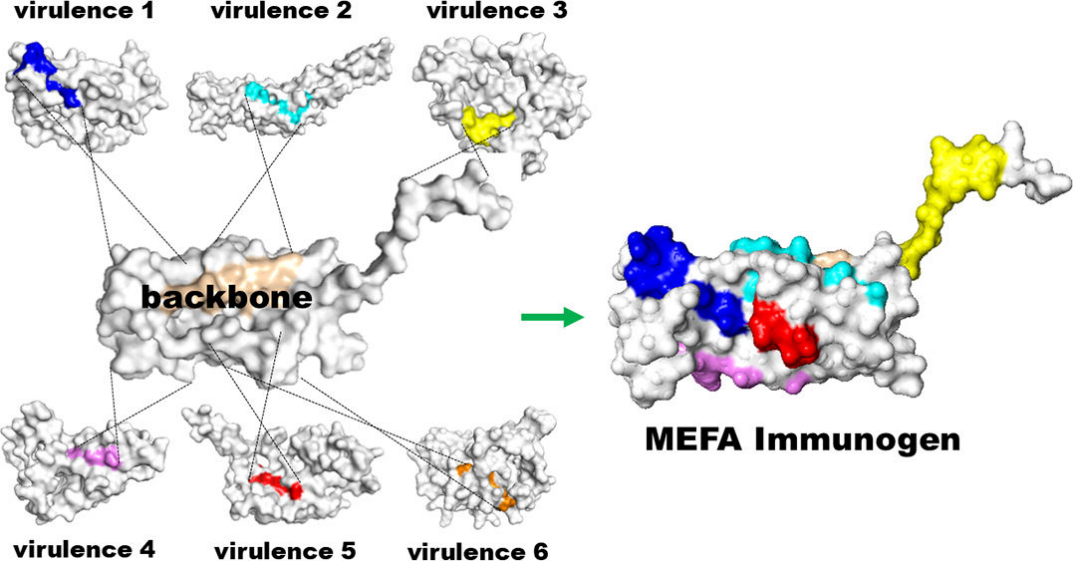
Conceptual scheme of multiepitope fusion antigen vaccinology platform. The MEFA platform utilizes a backbone immunogen to present foreign epitopes of interest and mimics the native antigenic propensity of the epitope for a polyvalent MEFA immunogen, thereby promoting broad immunity and cross-protection against heterogeneous virulence factors or strains.

This platform utilizes a backbone immunogen to present multiple immunodominant, and ideally functional, epitopes of different virulence determinants or several pathogenic strains and mimics epitope native antigenicity, thereby inducing broad protective immunity (Fig. 2). The backbone immunogen can be a universal molecule, a specific vaccine antigen, and/or an adjuvant. It should be non-toxic and non-allergenic, possess multiple surface-exposed and well-separated continuous epitopes (to be substituted with foreign epitopes of interest), have a stable structure (and remain stable after epitope substitution), and be easily expressed by a host strain, such as *E. coli* or another vaccine strain, preferably as a soluble protein at a relatively high yield. Ideally, this backbone carrier is thermally stable for an extended shelf life. The immunodominant or functional B-cell or T-cell epitopes representing the virulence determinants or pathogenic strains of interest can be obtained from epitope databases, predicted *in silico* using online software, or identified empirically. The epitope substitution can be optimized to avoid significant alteration or destabilization of the backbone structure and retain the native antigenic propensity of the foreign epitopes, aided by protein modeling and molecular dynamics simulation (62).

Advantages of the MEFA platform include (i) a strongly and broadly immunogenic polyvalent antigen and a cross-protective multivalent vaccine; (ii) a safer vaccine since it carries no potential harmful somatic antigens or components to cause adverse effects; (iii) precision and robust immune response as a MEFA-based vaccine precisely targets virulence determinants and prevents host immune system diffusion or frustration (from responding to non-target antigens); (iv) cost-effectiveness of manufacturing and simplification of formulation as only one immunogen or a few immunogens are involved, and if the MEFA immunogen is thermally stable, a cold chain is eliminated; (v) minimum interference from pre-existing antibodies (passive maternal antibodies or antibodies from pre-exposure to the target pathogens), since the vaccine targets one or two epitopes rather than a virulence determinant or a pathogenic organism; and (vi) the versatility of the platform, as it can be used to develop B-cell and T-cell epitope vaccines against bacterial, viral, and parasitic infections. The limitations include the requirement of computational skills, an adjuvant for an acellular vaccine if the MEFA immunogen is not self-adjuvanted, and a vector with high levels of expression and secretion of the MEFA immunogen.

We developed this MEFA platform at the beginning of the 2010s to generate a polyvalent ETEC adhesin MEFA immunogen. By substituting the surface-exposed and less immunodominant epitopes on the backbone, the major subunit (CfaB) of ETEC adhesin CFA/I, with the *in silico*-predicted most immunodominant B-cell epitopes from the major subunit of adhesins CS1 (CooA), CS2 (CotA), CS3 (CstH), CS4 (CsaB), CS5 (CsfA), and CS6 (CssA), we constructed the polyvalent CFA/I/II/IV MEFA protein. This ETEC adhesin MEFA immunogen induced broadly functional antibodies against the seven target adhesins (39) and reduced ETEC bacterial colonization by over two to three logs in rabbit small intestine (64). It was noted that the CFA/I/II/IV MEFA maintained a stable structure nearly identical to that of the backbone CfaB, based on protein modeling and molecular dynamics simulations (63). This MEFA protein also exhibited thermal stability, as it showed no visible degradation after being stored at 37°C for 6 weeks or 50°C for 4 weeks (96). These characteristics make CFA/I/II/IV MEFA an excellent antigen for an adhesin-based vaccine candidate or the adhesin antigen component of a multivalent ETEC vaccine (MecVax).

Our groups and others have applied the MEFA platform to construct polyvalent antigens for a cross-protective vaccine against other ETEC adhesins (40, 66, 101), *Shigella* spp. and serotypes (105), *Vibrio cholerae* serogroups (141), and heterogeneous ETEC strains associated with diarrhea in animals (142–146). Since 2014, the multiepitope vaccine concept has been applied in the research and development of cross-protective multivalent vaccines targeting different pathogens and diseases.

## CHALLENGE MODELS FOR ETEC VACCINE EVALUATION

Besides virulence heterogeneity, another major challenge in ETEC vaccine research and development is the lack of a suitable challenge model to evaluate the pre-clinical efficacy of ETEC vaccine candidates. An ideal animal model utilizes an animal species that is naturally susceptible to ETEC infection and, after infection, exhibits a clinical outcome similar to that of human patients. Equally important, these animals will develop immunity after vaccination or natural infection and become protected against subsequent homologous infection at the very least. Different challenge models have been attempted for ETEC vaccine development. That includes human, nonhuman primate, pig, rabbit, and mouse challenge models (Table 2).

**TABLE 2:** The infection models used in research and development of ETEC vaccine candidates

Infection model	Related property	Comment
Murine infection model	Preclinical efficacy against ETEC bacterial intestinal colonization	Mice are not naturally susceptible to ETEC infection. It requires antibiotic treatment to alter the mouse gut microflora.
Rabbit colonization model	Preclinical efficacy of active immunity against ETEC bacterial intestinal colonization	Rabbits are naturally susceptible to ETEC colonization in the small intestine, but they rarely develop diarrhea after ETEC infection.
Pig passive protection model	Preclinical efficacy of passive immunity against STa or LT toxin-mediated clinical diarrhea	Pigs are naturally susceptible to pig-type ETEC infection and develop clinical diarrhea. It requires immunizing pregnant mothers and challenging the newborn piglets with a recombinant ETEC strain that expresses STa or LT toxin.
A dual rabbit and pig model	Preclinical efficacy against ETEC bacterial intestinal colonization and clinical diarrhea	It combines the rabbit colonization model and the pig passive protection model to evaluate vaccine efficacy.
Nonhuman primate model	Preclinical efficacy against ETEC clinical diarrhea	*Aotus nancymaae* monkeys are naturally susceptible to ETEC infection and develop clinical diarrhea. They are expensive, with limited resources available.
Controlled human infection model	Clinical efficacy against ETEC diarrhea	This model can directly evaluate protection against ETEC diarrhea but is limited to the use of healthy adults.

### Murine infection model

Mice, the most commonly used laboratory animal, are unfortunately not naturally susceptible to ETEC infection and do not develop diarrhea after infection with ETEC. Without pretreatment with antibiotics to alter or deprive gut microflora, mice cannot be effectively colonized by ETEC in the small intestines. Antibiotic-treated mice have been used to examine protection against intestinal colonization by ETEC strain H10407 (69). However, the mouse infection model cannot assess the preclinical efficacy of vaccine candidates against ETEC diarrhea.

### Rabbit colonization model

Unlike mice, rabbits can be colonized naturally by ETEC bacteria. Thus, rabbits have been used to evaluate the preclinical efficacy of vaccines against ETEC bacterial colonization in the small intestine. Rabbits intramuscularly immunized with the ETEC vaccine candidate CFA/I/II/IV MEFA developed robust antigen-specific antibody responses. When challenged with the ETEC strain B7A, the immunized rabbits exhibited a two-log reduction in colonization by the challenge bacteria in the small intestine (64). Similarly, rabbits intramuscularly immunized with ETEC vaccine candidate MecVax developed antitoxin and anti-adhesin antibodies and showed a two- to three-log reduction of bacterial intestinal colonization when infected with the ETEC wild-type strains expressing CFA/I, CS1/CS3, CS2/CS3, CS3, CS4/CS6, CS5/CS6, or CS6 adhesins (100). However, adult rabbits do not develop diarrhea when given an orogastric challenge with ETEC. Thus, rabbits are primarily used to examine the immunogenicity and preclinical efficacy of ETEC vaccines against ETEC bacterial intestinal colonization.

### Pig passive protection model

Pigs, especially young pigs, are naturally susceptible to ETEC infection and develop clinical diarrhea nearly identical to that of children. This makes young pigs useful to evaluate ETEC vaccine efficacy against clinical diarrhea (24). This pig infection model has been used to evaluate protection against ETEC diarrhea for ETEC toxoid fusion vaccine candidate (45), adhesin-toxoid candidate (40), or MecVax (97). These pigs with vaccine-specific antibodies were protected against clinical diarrhea when challenged with an STa+ or an LT+ ETEC strain. The ETEC challenge strains, however, express a pig-specific type of adhesin since ETEC adhesins for humans and pigs are species-specific. Therefore, the ETEC challenge strains used in the pig infection model are recombinant; they express a pig-type ETEC adhesin and a human-type ETEC STa or LT (STa or LT; the human and pig ETEC strains equally stimulate intracellular cyclic guanosine or adenosine monophosphate or fluid accumulation in pig gut loops [147]). Unfortunately, pigs develop age-associated resistance to STa enterotoxicity (but not to LT toxin), likely due to the thickening of the mucin layer that covers the STa host receptor, guanylyl cyclase C (GC-C). Consequently, only young pigs develop clinical diarrhea after infection with an STa ETEC strain. Therefore, a passive protection pig model is used, in which pregnant sows are immunized, and the born piglets are challenged, demonstrating vaccine-specific passive antibody protection against ETEC STa- (or LT-) mediated clinical diarrhea.

### Rabbit and pig dual-challenge model

Since the rabbit infection model can be used to demonstrate preclinical efficacy against ETEC intestinal colonization, and the pig passive protection model can be used to indicate protection against STa or LT clinical diarrhea, by combining these two infection models, we can have a dual-animal challenge model to examine an ETEC vaccine candidate for protection against ETEC bacterial intestinal colonization and clinical diarrhea (97, 106).

### Non-human primate model

Unlike mice, rabbits, or pigs, nonhuman primates, particularly *Aotus nancymaae,* are naturally susceptible to ETEC bacterial intestinal colonization and clinical diarrhea. *Aotus nancymaae* developed strong immune responses after administration of an ETEC acellular vaccine candidate and was protected against subsequent challenges. This makes the *Aotus nancymaae* infection model, developed by the US NMRC, relevant for evaluating ETEC vaccine efficacy (52, 148, 149). This infection model has been used to examine the candidacy of the CFA/I tip adhesin (49, 51–53), tip adhesin and LT chimera (58), and a CS6-based product (60). The high cost, scarcity of the animal, and ethical concerns, however, essentially prevent the use of this non-human primate model for routine preclinical efficacy studies of ETEC vaccine candidates.

### Controlled human infection model

The controlled human infection model can be used to demonstrate vaccine protection by immunizing healthy adults with a candidate vaccine or placebo and then infecting them with the pathogen of interest in a highly controlled inpatient setting (150). By testing vaccine candidates directly in human subjects and selecting the promising ones, the CHIM can eliminate uncertainty or irrelevance from animal studies, particularly when suitable animal infection models are unavailable or inaccessible. After demonstrating safety and immunogenicity in a Phase-1 study, the CHIM accelerates the vaccine candidate down-selection process, helping to avoid the expense of field trials for less promising candidates. The use of CHIMs has been previously reviewed as a method to expedite ETEC vaccine development (147–150) and to characterize and identify ETEC challenge strains suitable for this model (151–153).

## CONCLUSION

Significant progress has been made over the last decade in advancing ETEC vaccine research and development, including noteworthy advances to facilitate the development of maximally effective ETEC vaccines. STa, one of the two ETEC toxins, can now be prepared as a safe and immunogenic antigen to provide coverage against its enterotoxic effects in ETEC infections. LT, the other toxin, has been detoxified and applied as a safe antigen to cover the LT enterotoxic effects and also a mucosal (and parenteral) adjuvant. Adjuvant dmLT was shown to significantly enhance immunogenic responses from ETVAX among infants and young children in LMICs, who generally respond poorly to vaccines orally administered. Integrating multiple heterogeneous antigens on a single backbone, the MEFA vaccinology strategy represents a significant step forward in broad vaccine protection coverage and enables the development of effective ETEC vaccine candidates.

Several new ETEC vaccine candidates have been demonstrated from preclinical studies to provide broad immunogenicity and cross-protection. Some existing cellular and acellular candidates have advanced to safety, immunogenicity, and/or efficacy studies in humans. In particular, the inactivated cellular candidate ETVAX has completed five Phase-1 studies and two Phase-2 studies, with two Phase-3 trials planned (112).

In this review article, we compiled a list of all potential ETEC vaccine candidates. However, we acknowledge that this list could be shortened, as some candidates, especially the monovalent products that focus on a single ETEC adhesin or toxin, such as CS6, LT, or STa, are unlikely to be finalized as an ETEC vaccine due to their limited protection coverage. Instead, these products may serve as additional antigens to be incorporated into another vaccine candidate for a multicomponent ETEC vaccine, providing broad protection. Even bivalent and multivalent candidates can be further combined for a new and more effective ETEC vaccine candidate if they are antigenically compatible; for example, toxoid fusion 3xSTa_N12S_-mnLT_R192G/L211A_ and CFA/I/II/IV MEFA, which could be individually developed as an antitoxin or anti-adhesin acellular vaccine candidate, respectively, were combined for a more protective ETEC vaccine candidate, MecVax.

Another concern for many candidates is their lack of an antigen to protect against STa toxin, which is more important in causing children’s and travelers’ diarrhea. Those STa-lacking candidates can be effective against LT ETEC infection but may be ineffective against ETEC diarrhea caused by STa-only strains. Recent progress using STa toxoid conjugates or fusion antigens to induce neutralizing or protective anti-STa antibodies should help overcome this challenge. Currently, MecVax and the *Shigella* and ETEC combined vaccine candidates, including ShigETEC, CVD *Shigella* and ETEC, and ShecVax, contain an STa antigen component. However, except for the acellular candidates (MecVax and ShecVax), the cellular STa-containing vaccine candidates have yet to be demonstrated to induce neutralizing anti-STa antibodies and protect against STa ETEC diarrhea.

Encouragingly, the new approach, which utilizes conserved antigens or polyvalent antigens constructed with a novel multiepitope fusion antigen vaccinology platform, expands vaccine coverage against infections from the most important, if not all, heterogeneous ETEC strains. Recently developed infection models, including suitable animal challenge models and the controlled human infection model, promise to accelerate ETEC vaccine research and development.
